# Multicentric pilot study to standardize clinical whole exome sequencing (WES) for cancer patients

**DOI:** 10.1038/s41698-023-00457-x

**Published:** 2023-10-20

**Authors:** Michael Menzel, Stephan Ossowski, Sebastian Kral, Patrick Metzger, Peter Horak, Ralf Marienfeld, Melanie Boerries, Steffen Wolter, Markus Ball, Olaf Neumann, Sorin Armeanu-Ebinger, Christopher Schroeder, Uta Matysiak, Hannah Goldschmid, Vincent Schipperges, Axel Fürstberger, Michael Allgäuer, Timo Eberhardt, Jakob Niewöhner, Andreas Blaumeiser, Carolin Ploeger, Tobias Bernd Haack, Timothy Kwang Yong Tay, Olga Kelemen, Thomas Pauli, Martina Kirchner, Klaus Kluck, Alexander Ott, Marcus Renner, Jakob Admard, Axel Gschwind, Silke Lassmann, Hans Kestler, Falko Fend, Anna Lena Illert, Martin Werner, Peter Möller, Thomas Theodor Werner Seufferlein, Nisar Malek, Peter Schirmacher, Stefan Fröhling, Daniel Kazdal, Jan Budczies, Albrecht Stenzinger

**Affiliations:** 1 Institute of Pathology, Heidelberg University Hospital, Heidelberg, Germany; 2 Center for Personalized Medicine (ZPM), Heidelberg, Germany; 3 Institute of Medical Genetics and Applied Genomics, University of Tübingen, Tübingen, Germany; 4 Center for Personalized Medicine (ZPM), Tübingen, Germany; 5 Institute for Bioinformatics and Medical Informatics (IBMI), University of Tübingen, Tübingen, Germany; 6 Institute for Surgical Pathology, Medical Center, University of Freiburg, Freiburg, Germany; 7 Center for Personalized Medicine (ZPM), Freiburg, Germany; 8 Institute of Medical Bioinformatics and Systems Medicine (IBSM), Medical Center – University of Freiburg, Faculty of Medicine, University of Freiburg, Freiburg, Germany; 9 Division of Translational Medical Oncology, German Cancer Research Center (DKFZ) and National Center for Tumor Diseases (NCT), Heidelberg, Germany; 10 German Cancer Consortium (DKTK), Heidelberg, Germany; 11 Institute of Pathology, University Hospital Ulm, Ulm, Germany; 12 Center for Personalized Medicine (ZPM), Ulm, Germany; 13 Comprehensive Cancer Center Freiburg (CCCF), Medical Center - University of Freiburg, Faculty of Medicine, University of Freiburg, Freiburg, Germany; 14 German Cancer Consortium (DKTK) Partner Site Freiburg, and German Cancer Research Center (DKFZ), Heidelberg, Germany; 15 Institute of Medical Systems Biology, Ulm University, Ulm, Germany; 16 Department of Anatomical Pathology, Singapore General Hospital, Singapore, Singapore; 17 Institute of Pathology and Neuropathology, University Hospital Tübingen, Tübingen, Germany; 18 Department of Medicine I, Medical Center-University of Freiburg, Faculty of Medicine, University of Freiburg, 79085 Freiburg, Germany; 19 Medical Department for Hematology and Oncology, Klinikum Rechts der Isar, Technische Universität München, 80333 Munich, Germany; 20 German Cancer Consortium (DKTK) Partner Site Munich, and German Cancer Research Center (DKFZ), Heidelberg, Germany; 21 Department of Internal Medicine I, University of Ulm, Ulm, Germany; 22 Department of Internal Medicine I, University Hospital Tübingen, Tübingen, Germany

**Keywords:** Biomarkers, Cancer genomics, Molecular medicine

## Abstract

A growing number of druggable targets and national initiatives for precision oncology necessitate broad genomic profiling for many cancer patients. Whole exome sequencing (WES) offers unbiased analysis of the entire coding sequence, segmentation-based detection of copy number alterations (CNAs), and accurate determination of complex biomarkers including tumor mutational burden (TMB), homologous recombination repair deficiency (HRD), and microsatellite instability (MSI). To assess the inter-institution variability of clinical WES, we performed a comparative pilot study between German Centers of Personalized Medicine (ZPMs) from five participating institutions. Tumor and matched normal DNA from 30 patients were analyzed using custom sequencing protocols and bioinformatic pipelines. Calling of somatic variants was highly concordant with a positive percentage agreement (PPA) between 91 and 95% and a positive predictive value (PPV) between 82 and 95% compared with a three-institution consensus and full agreement for 16 of 17 druggable targets. Explanations for deviations included low VAF or coverage, differing annotations, and different filter protocols. CNAs showed overall agreement in 76% for the genomic sequence with high wet-lab variability. Complex biomarkers correlated strongly between institutions (HRD: 0.79–1, TMB: 0.97–0.99) and all institutions agreed on microsatellite instability. This study will contribute to the development of quality control frameworks for comprehensive genomic profiling and sheds light onto parameters that require stringent standardization.

## Introduction

Scientific advances and technological developments over the past few decades led to a growing number of drugs that are available for treatment of cancer patients^1^. The paradigm of precision oncology, which is based on a mechanistic understanding of tumor biology and fine granular diagnostic profiling, is supported by the majority of clinical trials^2^. Furthermore, the clinical relevance of complex biomarkers, like microsatellite instability (MSI), tumor mutational burden (TMB), and homologous recombination deficiency (HRD), is increasing since they have been shown to be meaningful predictive biomarkers for patient stratification. As a result, there is movement towards more comprehensive DNA sequencing in routine clinical care, evident in the shift from the use of smaller (few to 100 genes) to larger (around 1 Mb) targeted panels in Next Generation Sequencing (NGS)^3–6^. While this diagnostic approach is generally supported by reimbursement schemes in national health care systems, whole exome sequencing (WES) and whole genome sequencing (WGS) are currently employed mainly in dedicated research programs aimed at comprehensive screening and identification of novel biomarkers and druggable targets^7–9^. But, with increasing sequencing capabilities and decreasing sequencing costs, WES and possibly even WGS have the potential to enter the clinical setting^10–15^. Compared to panel sequencing, WES offers the opportunity to (i) comprehensively cover the coding sequence so that either the entire exome or virtual gene panels can be investigated in the light of present and future diagnostic needs, (ii) facilitate comparability and standardization across different laboratories by avoiding bias of custom gene panels which may differ in design and require repetitive updates on gene content, and (iii) accurately measure (instead of estimate) complex biomarkers such as TMB, HRD, and MSI^16–19^.

WES focusing on cancer diagnostics has been investigated in various single center studies^9,12,20–22^, as well as in multi-center studies focusing on singular metrics including variant detection^23–28^, panel-based somatic variant detection^29–33^, CNAs^34–37^, or complex biomarkers^38–41^. However, WES studies that assess the use of real-world clinical tissue samples and investigate the reproducibility of diagnostic results across different diagnostic institutes covering the entire workflow from laboratory analysis to bioinformatic evaluation are scarce. To fill this gap, the Centers of Personalized Medicine of Baden-Wuerttemberg (ZPM)^42^ embarked on a joint pilot study to pave the way for the use of WES in routine cancer diagnostics.

Tumor and matched normal DNA samples from 30 patients covering a variety of cancer entities and molecular alteration types were distributed to four participating centers, located in Heidelberg, Freiburg, Ulm and Tübingen. The samples were sequenced in the respective molecular laboratories according to their protocols and analyzed using the local bioinformatic pipelines of five different institutions at the four ZPM partner sites. The institutions reported a set of pre-defined molecular biomarkers: somatic mutations and CNAs in a list of relevant genes as well as the complex biomarkers HRD, TMB, and MSI. Inter-institution comparisons showed a high concordance for the somatic variant calls, CNA calls and the three complex biomarkers. An in-depth analysis was performed to reveal the causes for differences between the results of the participating institutions.

## Results

**Fig. 1:**
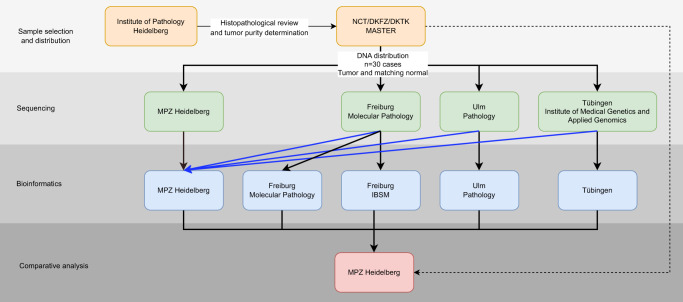
Overview on the study design. Tumor and normal DNA of 30 patients were distributed by DKFZ and NCT Heidelberg to four participating NGS laboratories. Bioinformatic analysis was performed at five participating departments. Additionally, all sequencing data were collected and analyzed with the same bioinformatic pipeline (blue arrows). Therapeutic relevant results from the DKFZ/NCT/DKTK MASTER program were collected and included in the comparative analysis.

Paired tumor and normal DNA samples from 30 patients were subjected to WES at four ZPM laboratories with subsequent data analysis performed at five ZPM bioinformatic institutions (Fig. 1). DNA was originally derived from fresh-frozen samples that had been analyzed by WES within the multicenter MASTER (Molecularly Aided Stratification for Tumor Eradication)^9^ program of the German Cancer Research Center (DKFZ), the National Center for Tumor Diseases (NCT) Heidelberg/Dresden, and the German Cancer Consortium (DKTK) before. The cohort consisted of a variety of rare cancer types including carcinomas, sarcomas, and other tumor entities selected to represent a wide variety of molecular alterations (Supplementary Table 1). Sequencing and bioinformatic analysis were performed agnostic of the tumor entity.

All DNA samples were successfully sequenced by all participating centers utilizing their local enrichment-kits, sequencers, and wet-lab protocols (Supplementary Table 2). Quality measures calculated for each of the sampels resuloted in on-target rates (percentage of bases in the interrogated region) of 41–83% and mean coverages after deduplication of 56–504x (Supplementary Figure 1). Concordance analysis between the five institutions of the four centers was performed in a predefined set of 494 oncological relevant genes (Supplementary Table 3).

### Somatic variant calling

**Fig. 2:**
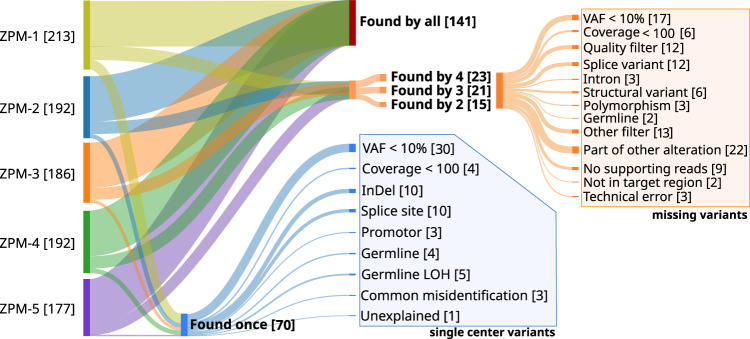
Inter-institution concordance of the detected somatic variants. Numbers in brackets refer to the numbers of somatic variants. Most variants were detected by all institutions (52%, red bar), while lower percentages of variants were detected by two to four institutions (22%, orange bar), and by only a single institution (26%, blue bar). Potential causes for discordant variant detection were analyzed and the variant sets were split accordingly.

Somatic variants were compared between institutions based on their chromosomal position and alteration at the DNA level. Only exact matches were accepted as the same variant. In the predefined gene set, a total of 960 somatic variant calls including 804 single-nucleotide variants (SNV) and 156 deletions/insertions were made by the five participating institutions. The variant calls corresponded to 270 unique variants of which the majority of 141 (52%) variants were detected by all five institutions, 59 (22%) unique variants were detected by two to four institutions, and 70 (26%) variants by a single institution (Fig. 2). We carried out an in-depth analysis of the discrepant variant calls by separately analyzing the variants that were detected by two to four institutions but missed by the remaining institutions (potentially false-negatives), and the variants detected by only a single institution (potentially false-positives).

Altogether, 59 unique variants were found by some but not all institutions (23 variants were found by four, 21 variants by three, and 15 variants by two institutions), corresponding to 110 missed findings of which 23 (21%) had a VAF < 10% or coverage < 100 (Fig. 2, orange subtree). For the remaining variants most misses were due to different filtering procedures including quality, PASS and other filters (37 missed variants, 34%). In addition, 22 (20%) variants were called, but annotated as part of other alterations. Only a small minority of 14 (13%) variant calls were missed due to the lack of supporting reads, technical errors, or not being covered by the target region. Two variant calls (2%) were falsely labeled as germline variant. Six (5%) variants were only identified as structural variants which were not assessed in this study. The remaining 6 (5%) variants were detected, but differently filtered due to intron location or labeling as polymorphism. The percentage of insertions/deletions (indels) missed by at least one of the centers (33%, CI 20–50%) was higher than the percentage of SNV missed by at least one center (20%, CI 15–26%).

Furthermore, 70 variants were called by only a single institution (Fig. 2, blue subtree). Out of these, 30 (43%) variants had VAF < 10% and 4 (6%) had a coverage < 100. Ten (14%) variants were splice site mutations that were detected by other institutions but classified as not affecting alternative splicing. Also, there were 10 (14%) insertions/deletions that were detected by only a single institution. The remaining variants were found in promoter regions (3 variants), were false-positives due to missed germline variants (4 variants), or a special case of loss of heterozygosity (LOH) as a somatic event (5 variants). Three variants were reported in the literature as common misidentified variants due to highly homologous genomic regions^43^, while only a single reported variant had no supporting reads in the data of the other institutions. The percentage of indels detected by only a single center (24%, CI 12–39%) was similar to the percentage of SNV detected by a single center (29%, CI 23–35%).

**Fig. 3:**
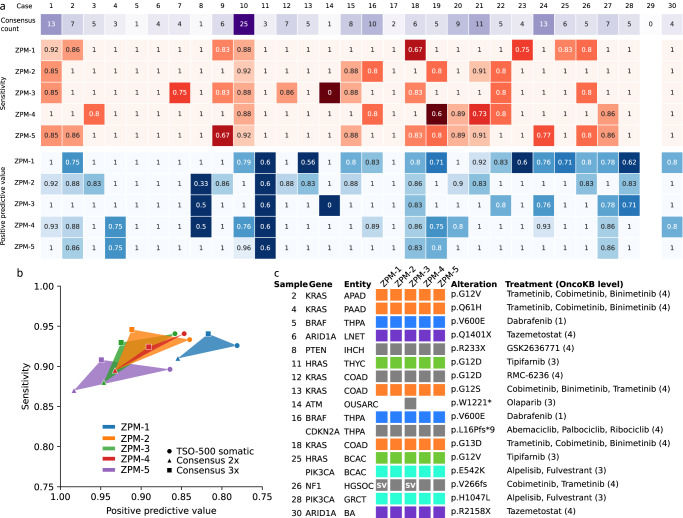
Comparison of the somatic variants detected by each of the institutions with a consensus list including all variants detected by at least three institutions. **a** Number of variants (3x consensus) for each of the cases as well as sensitivity and positive predictive value for each of the cases and institutions. Empty boxes (case 29) = no variants detected. **b** Sensitivity and positive predictive value for each of five institutions in comparison to three different references: consensus list of variants found in at least two institutions (Consensus 2x, triangle), consensus of variants found in at least three institutions (Consensus 3x, square), and TSO500 (circle). **c** Inter-institution concordance of therapeutic relevant somatic variants (OncoKB levels 1–4) with associated treatments and their OncoKB level. Boxes indicate detected variants and are colored by treatment option, variants marked with “SV” were found as structural variant.

Unfortunately, there is no ground truth that can serve as absolute reference for variant calling in clinical tissue samples. To address this limitation, the performance of the participating institutions was evaluated with respect to three different references: (i) a consensus list including all variants detected by at least three institutions (consensus 3x), and (ii) a consensus list including all variants detected by at least two institutions (consensus 2x), and (iii) the TSO500 assay that is used at our institution as validated laboratory developed test (LDT) for clinical mutation testing (Fig. 3a). When using the three-institution consensus as reference, all institutions achieved 100% sensitivity for 9 and at least 85% sensitivity for 16 of the 30 analyzed cases. Across the analyzed 30 cases, the five institutions achieved a positive percentage agreement (PPA) of 91–95% for the three-institution reference, 87–91% for the two-institution reference, and 90–94% for the TSO500 data reference (Fig. 3b). The corresponding PPV was in the range of 82–95%, 85–98%, and 78–86%, respectively (Fig. 3b).

### Therapeutically relevant variants

We carried out an in-depth analysis of variants in druggable genes according to OncoKB, evidence level 1–4^44^. Of 17 variants in druggable genes 16 were detected by all five institutions (Fig. 3c). The *NF-1* variant in sample 26 was reported by three institutions, while two institutions identified it as structural variant. The *ATM* variant in sample 14 was only detected by a single institution. We also compared the detected variants to the therapeutically relevant variants reported in the DKFZ/NCT/DKTK MASTER program (Supplementary Figure 2). The vast majority including 34 of 36 (94%) variants was detected by all institutions and the remaining two variants were found by all but one institution.

### Calling of somatic copy number alterations

**Fig. 4:**
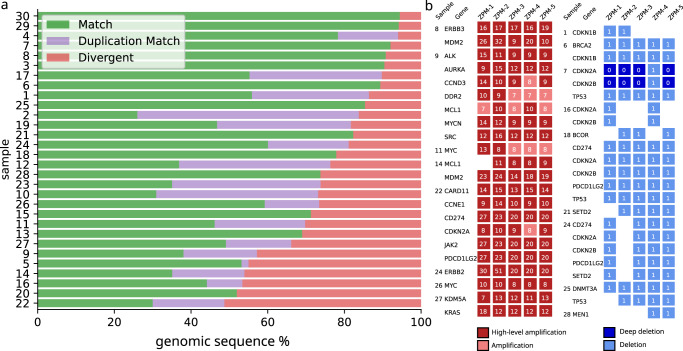
Inter-institution concordance of CNA calls. **a** Samples were compared between all pairs of institutions and the genomic sequence was split into segments with concordant CN (green), segments with concordant CN after correction for genome duplication (purple), and discordant CN (red). **b** Inter-institution concordance of amplifications and deletions in the set of oncogenic/likely oncogenic (according to OncoKB) genes. Colored box = CNA detected with number referring to the detected CN with amplifications in red and deletions in blue.

For each of the 30 tumors, segmentations of the genome in regions of constant copy number (CN) were compared pairwise between institutions. The genomic regions were classified as (i) exact matches, (ii) regions matching after considering genome duplication calls, and (iii) non-matching regions (Fig. 4). Summarizing the results for all 30 tumors, 76% of the genomic regions were matching exactly or after considering genome duplication calls (Fig. 4a, green/purple bars), while copy numbers were non-matching for 24% of the genomic regions (Fig. 4a, red bars). CN differences in divergent intervals were predominantly off by a CN of 1 (56%), followed by CN of 2 (26%), CN of 3 (11%), and higher CN (8%) (Supplementary Figure 3).

To distinguish between wet-lab variation and bioinformatic variability, we reanalyzed the data from all sequencing institutions using the same bioinformatic pipeline. Now 77% of the copy numbers were matching exactly or after considering genome duplications, while the remaining 23% of the copy number were non-matching (Supplementary Fig. 4). Thus, the result did hardly improve when harmonizing the bioinformatics and we can conclude that wet-lab variability was the main contributor to the observed inter-institution CN variability.

Tumor purity (determined by bioinformatics) appeared to be an important confounder for the determination of CNA: Cases with low tumor purity or variable estimates of tumor purity (cases 5, 16, 17, 20) had worse agreements between institutions (Supplementary Fig. 5).

### Therapeutic relevant gene amplifications and deletions

We carried out an in-depth analysis of all high-level (CN ≥ 5 delta from ploidy) amplifications (*n* = 22) and deep biallelic, as well as monoallelic gene deletions (*n* = 23) that were detected by at least two institutions (Fig. 4b). For 16 (73%) of the highly amplified genes, all institutions reported a high-level gene amplification. In five genes only two to four institutions reported a high-level amplification while the others reported an amplification below the high-level threshold. For the remaining single gene, four institutions reported a high-level amplification, while a single instution did not report any amplification. Eleven deletions (48%) were detected by all institutions of which two were deep deletions found by four institutions. Seven deletions were missed by a single institution, while the remaining five deletions were not reported by either two or three institutions. For sample 7, institution ZPM-4 detected deletion of *CDKN2A* and *CDKN2B* with CN = 1, while deep deletion of the two genes was detected by all other institutions. This discrepancy persisted when data processing was performed using a uniform bioinformatic pipeline meaning that it was caused by wet-lab variability.

### Complex biomarkers

**Fig. 5:**
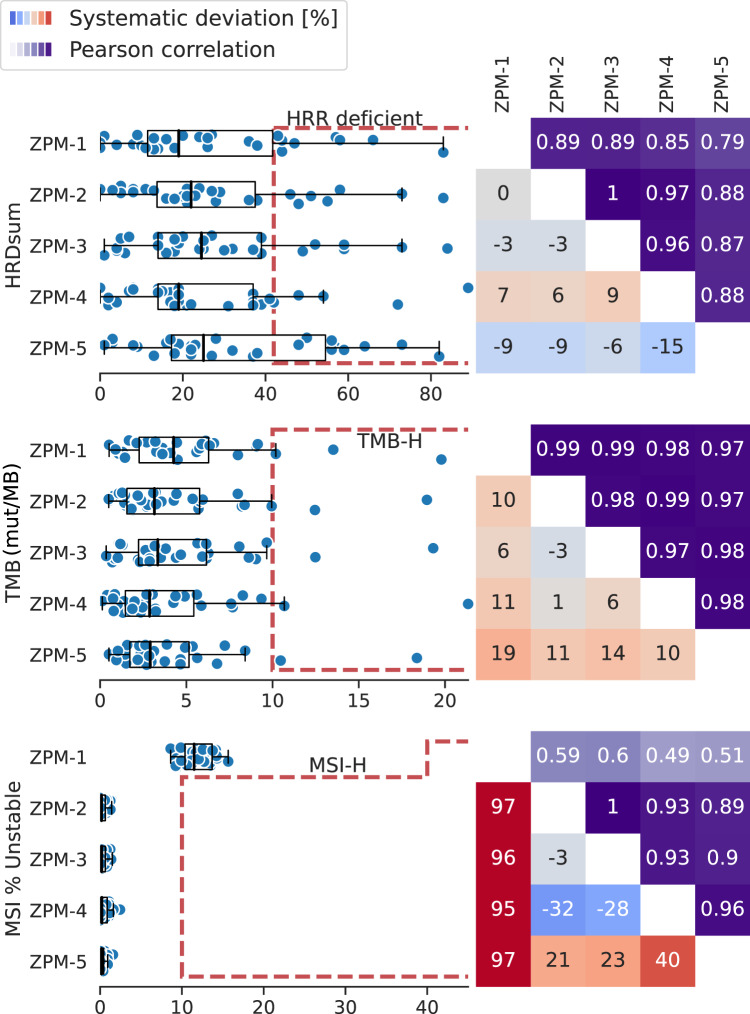
Inter-institution concordance of HRD, TMB, and MSI scores. Left: Distribution of the complex biomarkers scores together with the institution-specific cut-off points. Boxplots show the median, quartiles and whiskers up to 1.5 times of the box size. Right: Pearson correlation of the scores between pairs of institutions (upper triangle). Systematic deviation (in %) between two institutions (lower triangle). Red = systematic higher scores. Blue = systematic lower scores.

The HRD scores of the 30 tumors correlated strongly between the institutions (Pearson R between 0.79 and 1, Fig. 5). We observed systematic deviations between institutions in the range of −15% to +9%.

Four institutions used the same bioinformatic workflow (Sequenza^45^ and scarHRD^46^) and the same cut-off point of 42 for HRD calling, with one institution using a different segmentation tool (ClinCNV^47^). Different numbers of HRR-deficient tumors were detected: ZPM-4 reported five, ZPM-1 reported eight, ZPM-2 and ZPM-3 each seven, and ZPM-5 ten HRR-deficient cases. For five tumors, all institutions concordantly reported HRD (cases 9, 14, 15, 22, 26). For two additional tumors (cases 21 and 27), four institutions concordantly reported HRD, while the cut-off point was just missed by a single institution (HRDsum=37 and 39, both ZPM-4). In four cases (case 12, 16, 18, and 24) HRD was detected only by a single institution, once by ZPM-1 and three times by ZPM-5.

The TMB scores of the 30 tumors correlated strongly between the participating institutions (Pearson R between 0.97 and 0.99, Fig. 5). In-depth analyses showed a significant systematic deviation of institution ZPM-5 that reported 9–20% lower TMB scores compared to ZPM-1, ZPM-2, ZPM-3, and ZPM-4. Two of the tumors (cases 10 and 15) were identified as TMB-high by all institutions. Both were also reported as TMB-high in the DKFZ/NCT/DKTK MASTER program. Case 1 was reported as TMB-high by a single institution (TMB = 10.19) and close to the threshold of 10 mut/MB by the remaining four institutions (TMB between: 9.35–9.95). The remaining 27 tumors were concordantly reported as TMB-low by all institutions.

All tumors in the study cohort were MSS/MSI-L according to the NCT DKFZ/NCT/DKTK MASTER program. Accordingly, none of the institutions reported MSI-H for any of the tumors (Fig. 5). Four institutions used the same bioinformatic tool MSIsensor-pro^48^ resulting in a high concordance between MSI scores (Pearson R between 0.89 and 1). The usage of the tool Mantis^49^ by ZPM-1 together with a different cut-off point of 40% by ZPM-1 resulted in lower correlation of the MSI scores with the other institutions, but concordance concerning MSI status.

### Dissecting wet-lab and bioinformatic variability

To distinguish between wet-lab and bioinformatic variability, (i) sequencing data from three wet-labs were reanalyzed using the same bioinformatic pipeline and (ii) the sequencing data generated in Freiburg were analyzed by the bioinformatic pipelines of the Freiburg Institute of Pathology, the Freiburg ISBM, and the Heidelberg Institute of Pathology (Fig. 1).

**Fig. 6:**
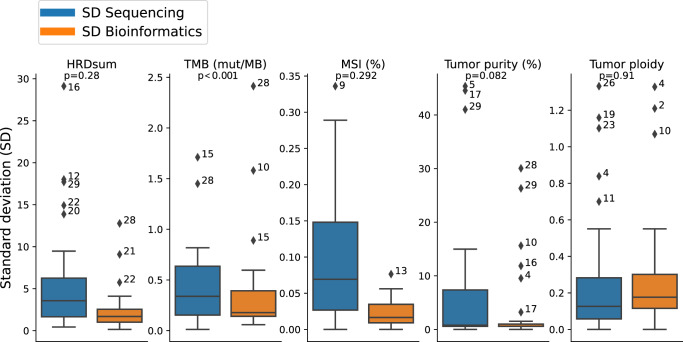
Comparison of bioinformatic and wet-lab inter-institution variability. To obtain “SD Bioinformatics”, the data of a single sequencing institution were evaluated by three bioinformatic institutions. To obtain “SD Sequencing” the data of three sequencing institutions were evaluated by a single bioinformatic institution. Outliers are annotated with the sample number. Boxplots show the median, quartiles and whiskers up to 1.5 times of the box size.

Results reveal that the median standard deviation (SD) separated for both wet-lab and bioinformatic effects is of relatively low influence for each biomarker (Fig. 6). For HRD, the median SD was 3.6 for the deviations by different wet-lab procedures and 1.7 for the deviations explained by different bioinformatics, both values being small compared to the cut-off point of 42. For TMB, the median SD was 0.34 mut/MB for the wet-lab and 0.18 mut/MB for the bioinformatics evaluation. Although the variability of TMB attributed to the wet-lab procedures was significantly higher than the bioinformatic variability, the former was still very low compared to the cut-off point of 10 mut/MB. For MSI scores, the median SD was 0.069% for the inter-wet-lab variabilty and 0.017% for the bioinformatic variability, both values being small compared to the cut-off point of 10%. For tumor purity, estimations also showed a very low median SD of 0.8% for the wet-lab divergence but showed outliers up to 45%, while the deviations in tumor purity estimation explained by bioinformatic variance had a median SD of 0.6% and outliers of up to 30%. For tumor ploidy, the median SD was similar for the variability explained by wet-lab difference (0.13) and for variability from bioinformatic processing (0.18).

For some of the samples, a high variability was observed simultaneously for several complex biomarkers. Cases 16, 28, and 29 showed high variability of HRDsum and simultaneously of the tumors purity estimations. As shown previously^50^, the influence of tumor purity and ploidy estimation on HRDsum scores calls for an accurate histological determination of tumor purity to improve the selection of a correct HRD score. Case 28 showed a high inter-wet-lab variability simultaneously for HRDsum, TMB, and tumor purity.

## Discussion

We performed a multicenter comparison of WES analysis of clinical cancer samples covering the entire sample-to-result workflow. This pilot study aimed to assess the level of concordance as well as to identify factors of inter-institutional variability. To this end, we analyzed (i) the inter-center concordance of the results and (ii) the dry lab as well as wet lab factors influencing the results. The study focused on three clinically relevant data layers, somatic variants, CNAs, and complex biomarker (HRD, TMB, MSI), and revealed high concordance between the participating institutions. However, we also identified areas of variability, notably the identification of CNAs. Collectively, these data provide a solid source and basis for standardization and harmonization of WES as a clinical-grade test and contribute to the conceptualization and design of future external quality assessment (EQA) schemes.

The majority of variants was detected by all institutions (52%) and almost all clinically relevant mutations (16 of 17) were consistently identified by the five participating institutions. In most cases the reasons for discrepancies could be identified, which are predominantly attributable to differences in quality and annotation-based filtering. Only a small fraction (1.5%) of consensus variants were missed due to shortcomings in sequencing or bioinformatic analysis. Our comparison of the WES data to the TSO500 panel derived data also showed that even mutations with lower VAF ( < 10%) could be identified in most institutions, yet are the most prominent reason for potential false-positive and false-negative calls. The clinically relevant *ATM* variant found by only one institute also showed low VAF (5.3%) and coverage (78X). In multi-center panel-based somatic variant calling studies detection rates of 80–100% were shown for clinically relevant alterations^29–32^. In one of the studies, the detection rate for SNV dropped to 82% when the restriction to clinically relevant variants was lifted^32^. In contrast to variant caller comparison studies^26,28^ where low concordance was observed, the downstream comparison utilized in our study improved results dramatically, even though a variety of variant callers was utilized (Supplementary Table 2). While detection rates of low frequency somatic variants are presumably biased using WES compared to panels due to reduced coverage, the advantages in routine diagnostics are profound. Alterations found in genes that are currently not clinically relevant can be reanalyzed in the future if required and complex biomarkers can be inferred directly from the same data.

Analysis stratified by variant type across the five participating centers showed that variants were missed more often for indels (33%) than for SNV (20%), while false positive variant detections were similar for both indels and SNV. These results are in line with earlier studies showing lower agreement of different variant callers for indels compared to SNV^24^. Thus, attention should be payed to implementation of a sensitive indel calling in bioinformatic pipelines to avoid missing of clinically relevant alterations such as for example activating indels in the exon 19 of the EGFR gene or truncating indels in tumor suppressor genes.

Compared to point mutations a larger variability was observed for CNA calling. High-level gene amplifications were found concordantly for most high-level amplifications calculated as delta from the ploidy. This raises the question as to which approach should be utilized. Gene deletions were shown to be disparate for several cases as a result of the previously described wet-lab variability. There is a lack of multi-center studies on CNA calling variability, but some reports indicating that the use of different bioinformatic tools for CNA detection is associated with significant variability^34,35^. However, we observed a high concordance of oncogenic/likely oncogenic amplifications and deletions between the utilized tools Sequenza (ZPM-2 to ZPM-5) and ClinCNV (ZPM-1) (Fig. 4b).

For the complex biomarkers HRD, TMB, and MSI, strong correlations of scores and high concordance of the diagnostic classification were observed even though bioinformatic pipelines were not harmonized prior to the pilot study. In contrast to previous studies performed using gene-panels, a higher concordance was observed for TMB^39,51,52^, which is attributable to the broader genomic footprint of WES. The high concordance of WES TMB values was also reported previously with correlations between 0.85 and 0.99 depending on tumor entity^20^. The same bioinformatic tools were used by all institutions for the determination of the HRD score. This leaves the observed variability attributed to wet-lab variance and configuration of bioinformatic tools. No multi-center study comparing HRD calls is currently known to the authors thus precluding consideration of the deviations in this context. Separation of bioinformatic and wet-lab variability allowed for an in-depth examination of inter-center variability leading to several lessons learned which will be discussed below.

Since the study focused on the entire sample-to-result workflow (result quality) including the laboratory analysis and the bioinformatic pipeline, we did not standardize wet-lab methods prior to the study (such as pre-analytics, library preparation, and sequencing). The study also did not specify the use of certain alignment or variant calling tools. This approach was taken intentionally to compare the performance of different NGS labs in the determination of clinically relevant somatic variants and genomic biomarkers in a real-world setting but is also a limitation of the study. A systematic comparison of different wet-lab methods, different variant callers or other bioinformatic tools was not intended and not feasible based on acquired data.

The study lacked a ground truth for the somatic variants, the CNA and complex biomarkers that could have been used as reference for comparison with the results of the participating institutions. Absence of a ground truth is unavoidable in comprehensive analysis of clinical tissue samples where a large number of genes are interrogated, but this is addressed in the current study by using a consensus reference and by comparison with deeper coverage panel sequencing data. Other studies have evaluated the performance of WES and WGS in samples with available ground truth but had to use artificial models such as cell lines^26,33^ or sequencing data with artificially added mutations to achieve this^25^, which does not reflect the clinical diagnostic setting. Taken together, the multitude of genetic events analyzed in WES coupled with the absence of a simply determinable ground truth when analyzing real-world clinical samples leads to a highly complex exercise compared to single-gene or few-gene tests which needs to be addressed conceptually by new kinds of proficiency tests.

The current study was performed using DNA extracted from fresh-frozen samples. Similar studies are warranted to evaluate the inter-center variability when analyzing formalin-fixed, paraffin-embedded (FFPE) samples, as low-confidence calls are more difficult to identify correctly in FFPE-material^53^. A further limiting factor for the generalization of the results was the location of all participating centers in Germany.

Based on our findings the following points should be considered for the implementation of WES as a clinical test in routine diagnostics: (i) Current clinically relevant alterations are identifiable with high precision and sensitivity despite non-harmonized wet lab and dry lab procedures. (ii) Detected somatic SNVs and indels show fairly high concordance with divergence mainly observed for low VAF variants in low-coverage regions, which were the main source of decreased sensitivity and PPV. Moreover, differences in the assessed region, annotation of variants in splice sites and promoter, and other quality or annotation-based filter criteria let to a small number of differentially reported variants, indicating the need for further harmonization of bioinformatics pipelines. (iii) Complex biomarker results show high concordance but are sensitive to issues with purity and ploidy estimation. (iv) High-level gene amplifications were reliably identified but other CNA (low level amplifications and deletions) were more difficult to detect primarily due to wet-lab variance and this needs to be reviewed accordingly.

In summary, calling of somatic variants was highly concordant with a PPA of 91–95% and a PPV of 82–95% compared against the three-institution consensus and full agreement for 16 of 17 druggable targets. Complex biomarkers correlated strongly between institutions (HRD: 0.79–1.00, TMB: 0.97–0.99) and all institutions agreed upon microsatellite stability status. Our data argue for the development of stringent standards defining a clinical-grade WES test and emphasize the need for harmonization of both wet lab and dry lab settings between institutions to ensure robust and comparable diagnostic results. The design of the current study and the strategies developed for the data evaluation will serve as a basis for a WES pilot involving 20 centers within the German Network for Personalized Medicine (DNPM^54^) and informs the development of future EQA schemes (e.g. in GenomDE^55^), including ring trials, for clinical-grade WES tests.

## Methods

### Case selection and distribution

Paired tumor and normal DNA samples from 30 patients were subjected to WES at four NGS laboratories and analyzed at five bioinformatic departments of the ZPM (Fig. 1). The 30 cases were selected from patients enrolled in the German DKFZ/NCT/DKTK MASTER program to represent a wide variety of molecular alterations. DKFZ/NCT/DKTK MASTER (NCT05852522) is a multicenter registry trial for prospective, biology-driven stratification of younger adults with advanced-stage cancer across all entities and patients with rare tumors. MASTER patients consented to banking of tumor and control tissue, molecular profiling of both samples, and clinical data collection (S-206/2011, Ethics Committee of the Medical Faculty of Heidelberg University). Tumor and germline DNA were shipped to each of the participating centers based on specific material transfer agreements. The cohort included a variety of rare cancer types including carcinomas, sarcomas, and other tumor entities, with a histopathologically determined tumor purity between 46% and 90% (Supplementary Table 1).

### Sequencing and data analysis

WES was performed independently by each laboratory using the established protocols with different sequencers, library kits, and sequencing chemistry. ZPM-1 and ZPM-5 generated sequencing libraries for tumor and normal samples using 100 ng of DNA and Twist Human Comprehensive Exome 2.0 (with additional custom regions), which were sequenced on Illumina NovaSeq6000 or NextSeq550Dx (see Supplementary Table 3). ZPM-4 also used the Twist Human Comprehensive Exome 2.0 sequenced on Illumina NovaSeq6000 with a varying amount of DNA for tumor and normal samples (Supplementary Table 3). ZPM-3 used the Agilent SureSelect XT Human All Exon V8 and sequenced on Illumina NextSeq 550 (Supplementary Table 3). Bioinformatic processing was performed independently in each institution using its custom pipeline (Supplementary Table 2). Quality control metrics were calculated using FastQC (https://www.bioinformatics.babraham.ac.uk/projects/fastqc/) and qualimap bamqc^56^ with each center using their target region as reference.

Furthermore, all tumor samples were re-analyzed using the Illumina TruSight Oncology 500 (TSO500)-panel. DNA integrity was assessed using the Genomic DNA ScreenTape Analysis on a 4200 TapeStation System (both Agilent, Santa Clara, California). To fragment DNA to a mean fragment size of around 200 bp, 80 ng DNA of each sample was sheared for 50 to 78 seconds using a focused ultrasonicator ME220 (Covaris, Woburn, Massachusetts). The library preparation for the capture-based TSO500 panel was performed according to the manufacturers’ manual with a final amplification of 15 cycles for the final libraries. Quality control and quantification was conducted using the KAPA SYBR Library Quantification Kit on a StepOnePlus quantitative PCR system (both Thermo Fisher Scientific). Up to eight libraries were sequenced simultaneously on a NextSeq 500 (Illumina) using high-output cartridge and v2 chemistry resulting in ~100 M read pairs (2 × 150 bp) per sample. All assays were performed according to the manufacturers’ protocols. DNA sequencing data was analyzed by TruSight Oncology 500 Local App (Illumina, pipeline version 2.2).

### Data collection and reference gene list

The following data were collected from the participating institutions in a predefined format: (1) variant calls, (2) copy number variation segments including allele-specific information, and (3) TMB, HRD, and MSI scores. The concordance of variant calls was evaluated in a list of 494 genes that included the American College of Medical Genetics (ACMG) list of cancer-related genes^57^ and genes utilized in the NCT MASTER program (Supplementary Table 3). Therapeutically relevant results for targetable genetic alterations reported in the NCT MASTER program were also acquired for each case.

Somatic variants were classified as therapeutically relevant if an annotation in level 1–4 of OncoKB of the corresponding entity or higher-level entity based on Oncotree^58^ was found.

Oncogenic deletions, oncogenic and likely oncogenic amplifications were downloaded from OncoKB^44^, and intersected with the segmented CNAs using BedTools^59^. Reported deep deletions and high-level amplifications (CN ≥ 5) were filtered based on the OncoKB list.

Variant calls from the TSO500 panel were filtered by the intersection of genes present in the gene list and panel (*n* = 274). As the TSO500 panel was used for sequencing of tumor DNA, germline variants were removed by subtracting the WES germline calls from all institutions combined.

### Bioinformatic versus sequencing variance

To distinguish between wet-lab and bioinformatic variability, raw sequencing data (FASTQ files) from all sequencing centers were re-analyzed using the same bioinformatic pipeline (Fig. 1, blue arrows). Conversely, raw sequencing data from the Freiburg sequencing center were analyzed using three different bioinformatic pipelines. As a result, wet-lab variability and bioinformatic variability could be evaluated separately. For the complex biomarkers, values were normalized by fitting a linear model with the intercept fixed at zero against the Heidelberg pipeline prior to calculation of the standard deviations. Statistical difference was assessed using the paired Wilcoxon test. *P*-values < 0.05 were considered significant.

### Inter-institution comparison of somatic variant, copy number, and complex biomarkers

Somatic variants were compared based on their chromosomal position, and alteration at the DNA level. Percentages of SNV and of indels were reported with 95% confidence intervals and calculated using the Clopper-Pearson method.

Copy numbers (CN) were collected as genomic intervals with position and respective allele-specific copy numbers. Regions without reported CN change were set to a CN of two. The proportions of the genome having CN = 0, 1, 2, 3, 4, … and the predominant ploidy were extracted. CN for each base of the genome were compared between each pair of two institutions. CN agreement was assessed as follows: (1) exact match, (2) discrepancy explained by the difference of the predominant ploidies (e.g., one institution reported a genome duplication, while the other institution did not), and (3) unexplained discrepancy.

HRD, TMB, and MSI scores were compared pairwise between institutions by correlations analysis (Pearson correlation) and by fitting of a linear model with the intercept fixed at zero. Statistical significance of difference was assessed with a one-sided Wilcoxon test and *p* < 0.05. All institutions used a cut-off point of 42 for HRD and a cut-off point of 10 mutations per megabase (MB) for TMB. For MSI, four institutions used a cut-off point of 10%, while a single institution used a cut-off point of 40% due to the application of a different bioinformatic approach for MSI detection (Supplementary Table 2). For sample 14 institution ZPM-1 was unable to determine the tumor purity and therefore correlations were calculated using 29 samples.

### Software for statistical analysis and graphics generation

Statistical analysis and figure creation were performed using Python with scipy^60^, pybedtools^61^, and numpy^62^. Figures were created using Matplotlib^63^, seaborn^64^, SankeyMatic (https://sankeymatic.com/), and Pandas^65^.

### Reporting summary

Further information on research design is available in the Nature Research Reporting Summary linked to this article.

### Supplementary information

Supplemental material

REPORTING SUMMARY

## Data Availability

The sequencing data generated in the study are available from the European Genome-phenome Archive (https://www.ebi.ac.uk/ega/datasets) under the Accession Number EGAD00001011087.

## Code availability

The code used to compare the results of the five participating institutions is available upon request to the corresponding authors.
