# Supplementary material for: Multicentric pilot study to standardize clinical whole exome sequencing (WES) for cancer patients
Source: NPJ Precis Oncol. 2023 Oct 20;7:106. doi: 10.1038/s41698-023-00457-x (PMC10589320; doi:10.1038/s41698-023-00457-x)
Supplement: Supplementary file 1 — Supplemental material [file 41698_2023_457_MOESM1_ESM.pdf]

Supplementary Figure 1

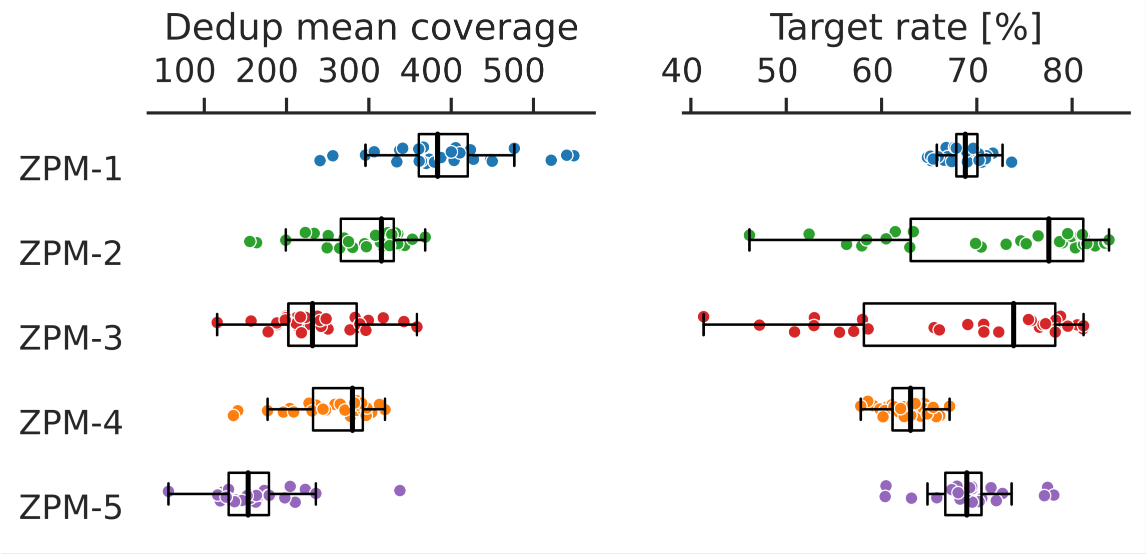

Quality measurements of the participating institutions with on-target rates and mean coverages after duplication for each sample. Boxplots show the median, upper and lower quartiles and whiskers up to 1.5 times of the box size.

Supplementary Figure 2

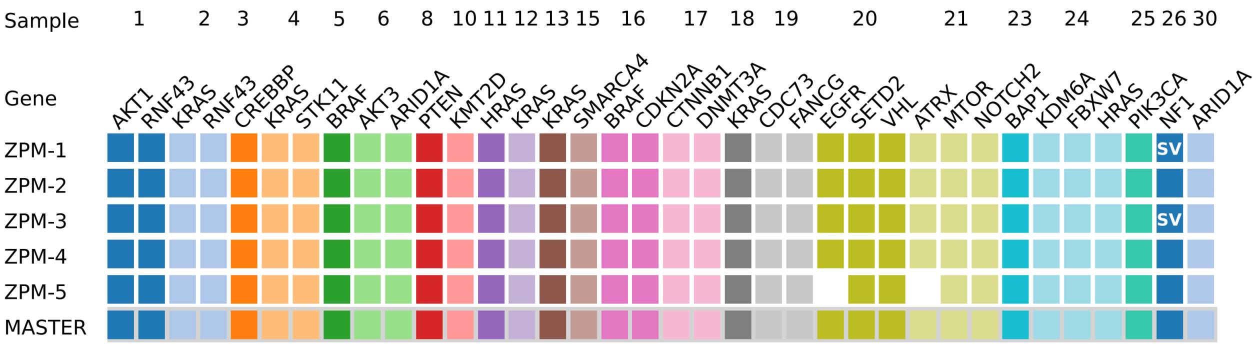

Comparison of therapeutically relevant variants reported in the DKFZ/NCT/DKTK MASTER program to variants found by the participating institutions.

Supplementary Figure 3

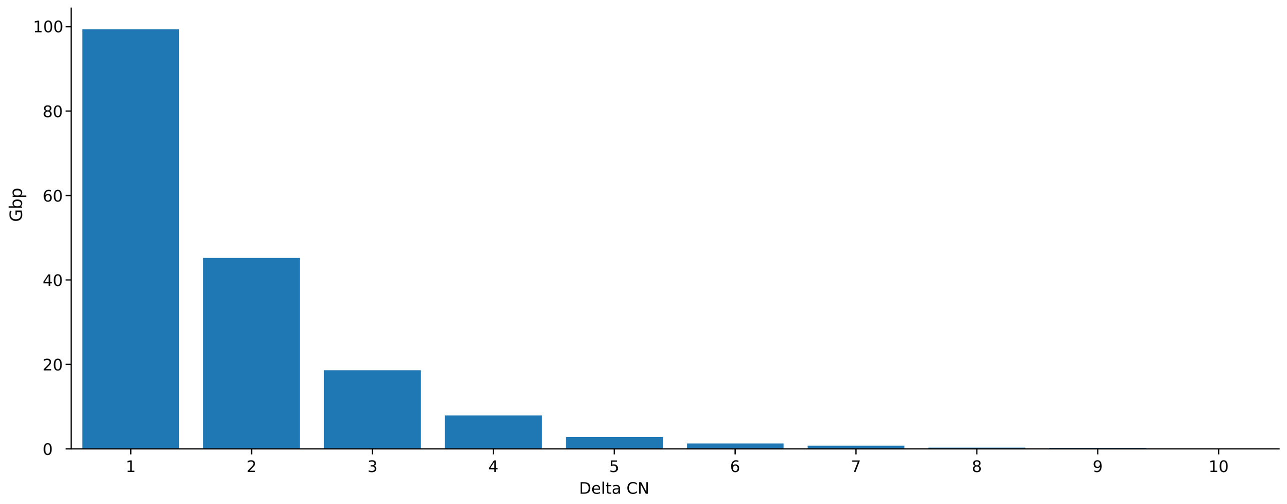

Differences of copy numbers between institutions for non-matching genomic regions.

Supplementary Figure 4

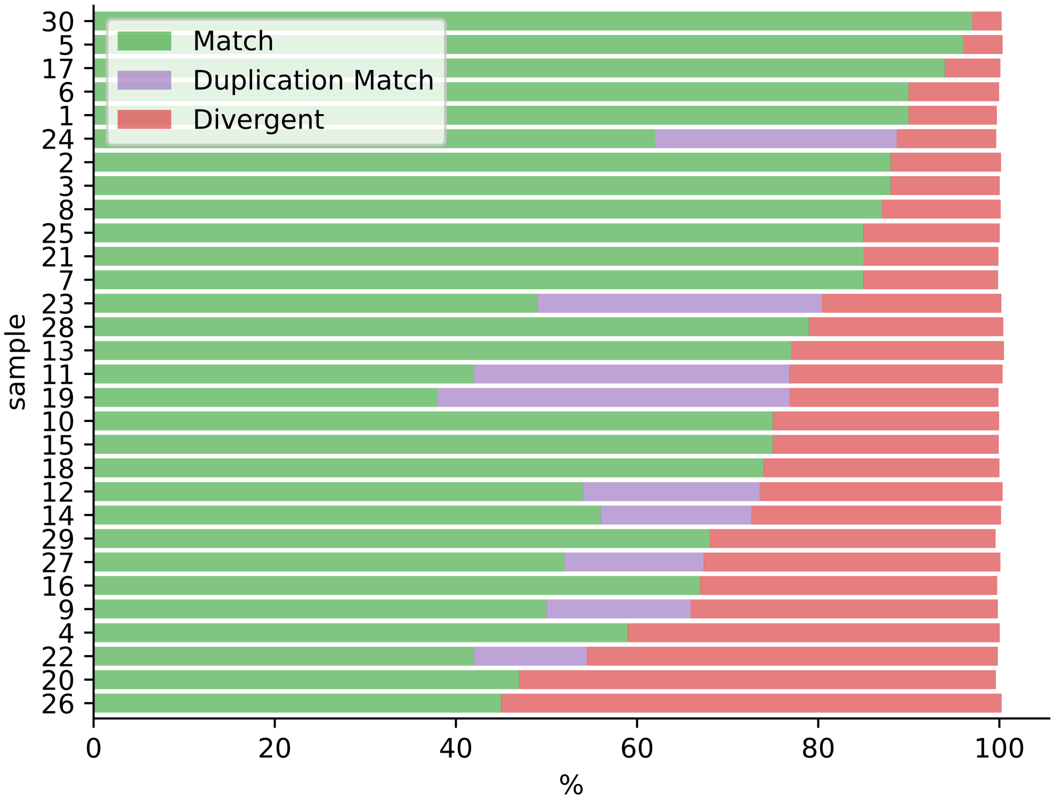

Analysis of copy number calls from all institutions using the same pipeline. In comparison to Figure 4 a no significanct improvement is observed by using the same pipeline.

Supplementary Figure 5

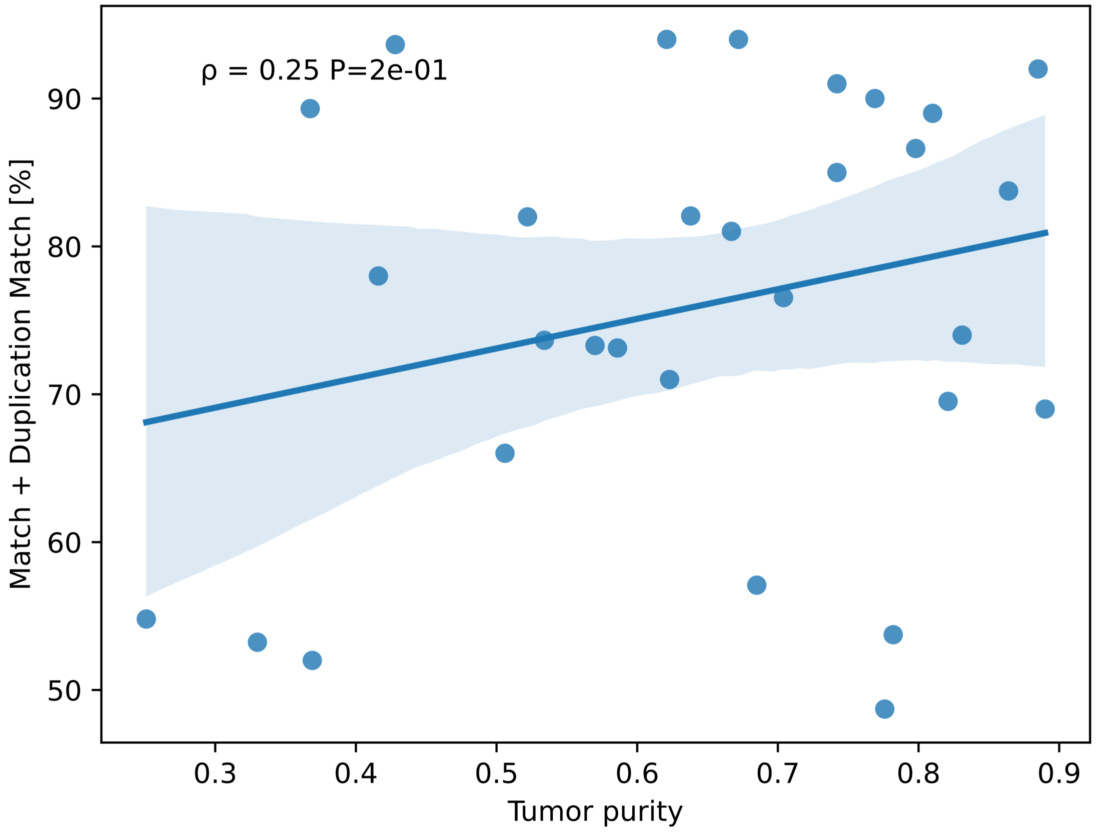

Agreement of copy number changes in contrast to tumor purity. The correlation shows an increased agreement for samples with high tumor purity.

Supplementary Table 1

Pseudonym	Entity	Tumor purity (Pathologist)
ZPMWES-T1	Adenocarcinoma of the appendix	64
ZPMWES-T10	Parathyroid carcinoma	81
ZPMWES-T11	Thymic squamous cell carcinoma	56
ZPMWES-T12	Adenocarcinoma of the rectum	68
ZPMWES-T13	Adenocarcinoma of the colon	54
ZPMWES-T14	Carcinosarcoma (MMMT) of the uterus	81
ZPMWES-T15	Adenocarcinoma of the lung	54
ZPMWES-T16	Papillary thyroid carcinoma	55
ZPMWES-T17	Desmoid fibromatosis	80
ZPMWES-T18	Adenocarcinoma of the colon	50
ZPMWES-T19	Parathyroid carcinoma	60
ZPMWES-T2	Adenocarcinoma of the appendix	72
ZPMWES-T20	Sarcomatoid renal cell carcinoma	81
ZPMWES-T21	Parathyroid carcinoma	52
ZPMWES-T22	Sarcoma, NOS	55
ZPMWES-T23	Epithelioid pleural mesothelioma	54
ZPMWES-T24	Salivary duct carcinoma of the parotid	52
ZPMWES-T25	Basal cell adenocarcinoma of the parotid	75
ZPMWES-T26	High grade serous carcinoma of the ovary	56
ZPMWES-T27	Nonseminomatous mixed germ cell tumor of the testis	76
ZPMWES-T28	Adult granulosa cell tumor malignant	76
ZPMWES-T29	NUT carcinoma of the paranasal sinus	63
ZPMWES-T3	Juvenile granulosa cell tumor of the ovary	46
ZPMWES-T30	Angiosarcoma of the breast	55
ZPMWES-T4	Ductal adenocarcinoma of the pancreas	58
ZPMWES-T5	Papillary thyroid carcinoma	76
ZPMWES-T6	Neuroendocrine tumor (NET, G2) of the lung	59
ZPMWES-T7	Thymoma	56
ZPMWES-T8	Intrahepatic cholangiocarcinoma	52
ZPMWES-T9	Mixed germ cell tumor of the testis	90

Supplementary Table 2

	ZPM-1	ZPM-3	ZPM-4	ZPM-5
**Case**	**DNA [ng]**	**DNA [ng]**	**DNA [ng]**	**DNA [ng] T/N**
1	100	100	178	100/100
2	100	100	116	100/100
3	100	100	37	100/100
4	100	100	118	100/100
5	100	100	116	100/100
6	100	100	84	100/100
7	100	100	120	100/100
8	100	100	108	100/100
9	100	100	112	100/100
10	100	100	110	100/100
11	100	100	106	100/100
12	100	100	112	100/100
13	100	100	112	100/100
14	100	100	106	100/100
15	100	100	106	100/100
16	100	100	104	100/100
17	100	100	157	100/100
18	100	100	110	100/100
19	100	100	112	100/100
20	100	100	165	100/100
21	100	100	110	100/100
22	100	100	56	100/100
23	100	100	116	100/100
24	100	100	114	100/100
25	100	100	110	100/100
26	100	100	114	100/100
27	100	100	108	100/100
28	100	100	110	100/100
29	100	100	114	100/100
30	100	100	113	100/100
**Manufacturer sequencer**	Illumina	Illumina	Illumina	Illumina
**System name**	Novaseq 6000	NextSeq 550	Novaseq 6000	Novaseq 6000 / Nextseq 550Dx
**Flowcell**	S2	High Output	SP	SP / S2 / High Output
**Cycles**	200	300	200	149
**Library**	Twist (Twist EF Library Prep 2.0)	Agilent	Twist	Twist
**Manufacturer chemistry**	Illumina NovaSeq 6000 S2 Rgt Kit v1.5 (200cyc)	NextSeq 500/550 HighOutput Kit v2.5 (300 Cycles), Cat# 20024908, Illumina	Twist	Twist
**Enrichment Kit**	Twist Standard Hyb Kit, Twist Comprehensive Exome + Custom Panel	SureSelect XT Human All Exon V8	Twist Exome 2.0	Twist Exome 2.0
**Enrichment Kit Catalog Number**	Design ID: TE- 94167158 -20°C 101042<br><br>Twist Standard Hybridization and Wash Kit v1: 104178 (12rxn)<br>Twist EF Library Prep 2.0: 104207 (96rxn)<br><br>Twist Hybridization Reagents (Box1) -20°C<br>Twist Wash Buffers (Box2) 2-8°C<br>Twist Universal Blockers: 100578 (12rxn)<br>100767 (96rxn)<br>Universal Blockers & Blocker Solution -20°C<br>Twist Binding and Purification Beads: 100983 (12rxn)<br>100984 (96rxn)<br>Streptavidin Binding Beads<br>DNA Purification Beads 2-8°C<br>NEBNext® Ultra™ II Q5® Master Mix -20°C M0544<br>Q5® High-Fidelity DNA Polymerase -20°C M0491	SSELXT Human All Exon V8, Cat. # 5191-6891, Agilent	Twist Universal Blockers, 12rxn, 100578<br>Twist Std Hyb and Wash Kit v2 with amp mix, 12rxn, 105560<br>Twist Dry Down Beads, 12rxn, 104325	TWIST Library Preparation Kit, Mechanical Fragmentation with amp Mix, 96 rxn 10477 1x<br>TWIST Universal Adapter System Plate A 96 well 101308 1x<br>TWIST Universal Blockers, 12 rxn 100578 1x<br>Twist Hybridization and Wash Kit mit Amplifikationsmix, 12 rxn 104178 1x<br>TWIST Dry Down Beads 12 rxn 104325 1x<br>TWIST Comprehensive Exome 12 rxn 102032 1x
**Sample Barcodes**	Twist Universal Adapter System (AD153 Barcode Product Number 30-05150-00)	SureSelect XT HS2 Index Primer Pairs for ILM	IDT Adapter	Twist Adapter
**Adapter system**	Twist Universal Adapter System	Universal adapter system (TruSeq)	Univeral adapter system	Twist Adapter System
**Library Prep Kit**	Twist Universal Adapter System TruSeq-kompatibel, 16 Proben -20°C 101307<br>Platte A -20°C 101308 Platte B -20°C 101309 Platte C -20°C 101310 Platte D -20°C 101311	SureSelect XT HS2 DNA Reagent Kit with Index Primer Pairs, Cat# G9983A, Agilent	Twist Library Preparation Kit, Mechanical, Fragmentation with amp mix, 96rxn, 104177<br>Twist UMI Adapter System - TruSeq Comp, Sample Plate A, 96well, 105041<br>Twist Exome2.0, +CompExome spike-in, 12rxn, 105229	TWIST Library Preparation Kit, Mechanical Fragmentation with amp Mix, 96 rxn 10477<br>TWIST Universal Adapter System Plate A 96 well 101308<br>TWIST Universal Blockers, 12 rxn 100578<br>Twist Hybridization and Wash Kit mit Amplifikationsmix, 12 rxn 104178<br>TWIST Dry Down Beads 12 rxn 104325<br>TWIST Comprehensive Exome 12 rxn 102032
**Target regions**	Twist Comprehensive Exome + Custom Panel	SureSelect XT Human All Exon V8 (hg19)	Twist Exome 2.0	Twist Exome 2.0
**UMIs**	No	Yes	No	No

Supplementary Table 2

			ZPM-1	ZPM-2	ZPM-3	ZPM-4	ZPM-5
		**Genom**	hg19 (liftover)	hg19	hg19	hg19	hg19
Biomarker	TMB	**Coverage**	20 (3 variant reads)	8x (4 Varianten Reads)	50	100	100
		**Counting**	SNV + INDEL	SNV + INDEL	SNV + INDEL	SNV + INDEL	SVN + INDEL
		**VAF threshold**	0.05 in tumor && < 0.17*tumor_freq in normal	0.05	0.05	0.035	0.05
		**Exone_filter**	FALSE	TRUE	TRUE	TRUE	TRUE
		**Region coverage**	20	50	50	100	100
	MSI	**Tool**	Mantis	Msisensor-pro	Msisensor-pro	Msisensor-pro	Msisensor-pro
		**Cutoff**	40%	10%	10%	10%	10%
		**MSI parameter**	-mrq 20 -mlq 25 -mlc 20 -mrr 1	-c 20	default parameters	-c 20	-c20 -f0.05 -q3
	HRD	**Segmentation**	ClinCNV	Sequenza	Sequenza	Sequenza	Sequenza
		**Segmentation parameter**	--colNum 4 --lengthS 9 --scoreS 200 --filterStep 2 --hg38 --reanalyseCohort FALSE	-w 50	default parameters		sequenza-utils bam2seqz -gc hg19gc50base.wiggle (gc_wiggle -w50 --> gc50base-wiggle --> -gc hg19gc50base)
		**Scoring**	scarHRD	scarHRD	scarHRD	scarHRD	scarHRD
		**Scoring parameter**			default parameters		sequenza-result, ploidy0=ploidy, sizelimitLOH=15e6
Somatic variants		**Variantcaller**	Strelka2	VarScan2 + Mutect2	Manta + Strelka2	Dragen	Mutect2
		**Variantcaller parameter**	--exome --indelCandidates [indelCandidates.vcf.gz]	--min-coverage 8 (VarScan2);<br>--callable-depth 8 (Mutect2)	-- CallRegion Agilent S33266340<br>--exome --indelCandidates	--vc-callability-tumor-thres 20	--minimumMappingQuality=1 –minBaseQualityScore=10,<br>--intervalPadding=50
		**VAF threshold**	0.05 in tumor && < 0.17*tumor_freq in normal	0.05	0.05	0.035 / 0.05	0.05
		**Basequality**		28	20		25
		**Coverage threshold**	20 (3 variant reads)	8x (4 Varianten Reads)	50	50 – 200	10
		**Annotation**	VEP	ANNOVAR + snpEff	ANNOVAR	ANNOVAR + snpEff	VEP
CNV		**Tool**	ClinCNV	Control-FREEC	Sequenza	Sequenza	Sequenza
		**CNV parameter**	--colNum 4 --lengthS 9 --scoreS 200 --filterStep 2<br>--hg38 --reanalyseCohort FALSE	breakPointType: 4; breakPointThreshold: 1.2;<br>MinCNAlength: 3; readCountTreshold: 50; window: 0	ploidy = seq(1, 5.5, 0.1) ;<br>Female=TRUE/FALSE		sequenza-utils bam2seqz -gc hg19gc50base.wiggle<br>(gc_wiggle -w50 --> gc50base-wiggle --> -gc hg19gc50base)

Supplementary Table 3

ABCB1	CAMTA1	CRKL	ERCC6	GATA2	JUN	MMS19	OSM	PRKAR1A	RSPO3	TEK
ABCB5	CARD11	CRTC1	ERG	GLI1	KDM5A	MN1	OSMR	PTCH1	RUNX1	TERT
ABL1	CBL	CSF1R	ERRFI1	GLI2	KDM5C	MPL	P3H1	PTCH2	SBDS	TET1
ABL2	CCND1	CSF2RA	ESR1	GLI3	KDM6A	MRE11	PAK4	PTEN	SDHA	TET2
ABRAXAS1	CCND2	CSF3R	ETS1	GNA11	KDR	MS4A1	PALB2	PTGFR	SDHAF2	TFE3
ADORA2A	CCND3	CTAG1B	EWSR1	GNAQ	KIF1B	MSH2	PARP1	PTPN1	SDHB	TGFB1
AIP	CCNE1	CTLA4	EXT1	GNAS	KIR2DL1	MSH6	PARP2	PTPN11	SDHC	TMEM127
AKT1	CCR2	CTNNB1	EXT2	GPC3	KIR2DL2	MSLN	PARP3	PTPN12	SDHD	TMPRSS2
AKT2	CCR4	CXCL12	EZH2	GPRC5A	KIR2DL3	MST1R	PAX3	PTPN2	SEM1	TMSB4X
AKT3	CCR5	CXCR4	FAM30A	GRM1	KIT	MTOR	PAX5	PTPRF	SETD2	TNFAIP3
ALK	CD19	CYLD	FANCA	GSTM1	KITLG	MUTYH	PAX7	PTPRJ	SF3B1	TNFRSF18
APC	CD27	CYP3A5	FANCB	GSTT1	KLB	MYB	PBRM1	PTPRK	SHH	TNFRSF4
AR	CD274	DAXX	FANCC	H3F3A	KMT2A	MYC	PDCD1	RAB35	SLC39A6	TNFRSF9
ARAF	CD276	DDB1	FANCD2	HAVCR2	KMT2B	MYCL1	PDCD1LG2	RAD18	SLFN11	TP53
ARHGAP26	CD40	DDB2	FANCE	HDAC2	KMT2C	MYCN	PDGFA	RAD50	SLX4	TPTE
ARHGAP6	CD5	DDIT3	FANCF	HEY1	KMT2D	MYCT1	PDGFB	RAD51	SMAD4	TRAP1
ARHGEF12	CD70	DDR1	FANCG	HGF	KRAS	MYD88	PDGFRA	RAD51AP1	SMARCA4	TRIM37
ARID1A	CD79B	DDR2	FANCI	HLA-A	KSR2	MYEOV	PDGFRB	RAD51B	SMARCAL1	TSC1
ASCL1	CD99	DICER1	FANCL	HORMAD1	LAG3	MYOD1	PELP1	RAD51C	SMARCB1	TSC2
ASPSCR1	CDC73	DIS3L2	FANCM	HORMAD2	LATS1	NAB2	PHF1	RAD51D	SMARCE1	TSHR
ATM	CDH1	DLEU1	FAS	HPS1	LCK	NAMPT	PHF6	RAD54B	SMO	TSSK3
ATR	CDH17	DLL3	FASLG	HRAS	LIG1	NBN	PHLPP1	RAD54L	SOCS1	TYR
ATRX	CDK1	DNMT3A	FBXW7	HSP90AA1	LMTK3	NCOA2	PHOX2B	RAF1	SOX10	UBE2N
AURKA	CDK12	DOT1L	FGF1	HSP90AB1	LPP	NDRG1	PIK3C2G	RAP1B	SPTA1	USP11
AURKC	CDK2	DUX4	FGF2	HSP90B1	LZTR1	NF1	PIK3CA	RARA	SRC	USP6
AXL	CDK4	EGF	FGF3	ICOS	MAGEA3	NF2	PIK3CD	RASA1	SRSF2	VEGFA
B2M	CDK5	EGFL7	FGF4	ID2	MALAT1	NFATC1	PIK3R1	RASA2	SS18	VEGFB
BAP1	CDK6	EGFR	FGFR1	ID3	MAML2	NFIB	PIK3R2	RB1	SSRP1	VHL
BARD1	CDK9	EGLN1	FGFR2	IDH1	MAP2K1	NFKBIA	PIK3R5	RECQL	SSTR1	VSIR
BCL2	CDKN1A	EGLN2	FGFR3	IDH2	MAP2K2	NFKBIE	PIM1	RECQL4	SSTR2	WIF1
BCL3	CDKN1B	EHBP1	FGFR4	IDO1	MAP3K1	NOTCH1	PIM2	RET	SSTR3	WNK2
BCL6	CDKN1C	EP300	FH	IDO2	MAP3K6	NOTCH2	PLAG1	RHBDF2	SSTR4	WRN
BCL9	CDKN2A	EPAS1	FLCN	IGF1R	MAX	NR4A3	PLK1	RHEB	SSTR5	WT1
BCOR	CDKN2B	EPCAM	FLI1	IKZF1	MCL1	NRAS	PLK2	RHOA	SSX1	WWTR1
BLM	CDKN2C	EPHA3	FLT1	IL21R	MDH2	NRG1	PLK3	RICTOR	SSX2	XAB2
BMPR1A	CDKN2D	EPHA5	FLT3	IL6	MDM2	NRG2	PMEL	RINT1	STAT3	XPA
BRAF	CEBPA	EPHB2	FLT4	IL6R	MDM4	NRG3	PMS1	RIT1	STAT5B	XPC
BRCA1	CEP57	ERBB2	FOLH1	IL6ST	MED12	NRG4	PMS2	RNF2	STAT6	XPO1
BRCA2	CHEK1	ERBB3	FOXL2	IL7R	MEN1	NSD1	PNKP	RNF43	STK11	XRCC1
BRD2	CHEK2	ERBB4	FOXO1	IRF4	MERTK	NT5E	POLD1	ROR1	STK36	XRCC2
BRD3	CIC	ERCC1	FOXP1	IRS2	MET	NTRK1	POLE	ROS1	SUFU	XRCC3
BRD4	CLDN18	ERCC2	FUS	JAK1	MITF	NTRK2	PPM1D	RPA1	SUZ12	YAP1
BRIP1	CLDN6	ERCC3	FYN	JAK2	MLH1	NTRK3	PRAME	RPTOR	SYK	YWHAE
BTK	CREBBP	ERCC4	GAK	JAK3	MLH3	NUDT1	PRF1	RRAS2	TACSTD2	ZNF217
BUB1B	CREBRF	ERCC5	GAS6	JAZF1	MLLT1	NUTM1	PRKACA	RSPO2	TEAD1	
